# A Novel Approach to Assessing and Conservatively Treating Anterior Cutaneous Nerve Entrapment Syndrome: A Case Study

**DOI:** 10.7759/cureus.44912

**Published:** 2023-09-08

**Authors:** David P Newman, Saige M Holkup, Erica L Masi, Adam T Soto

**Affiliations:** 1 Pain Management-Physiotherapy, Tripler Army Medical Center, Honolulu, USA; 2 Biology, Harvard University, Cambridge, USA; 3 Pain Management, Tripler Army Medical Center, Honolulu, USA; 4 Anesthesiology, Pain Management, Alexander T. Augusta Military Medical Center, Fort Belvoir, USA

**Keywords:** myofascial pain, manipulative, physical therapy, lower quadrant pain, abdominal cutaneous nerve entrapment

## Abstract

Anterior cutaneous nerve entrapment syndrome (ACNES) is a common source of chronic abdominal pain and is often underdiagnosed despite numerous and potentially invasive diagnostic evaluations and tests. We present a case report describing a novel, conservative, and non-invasive approach to diagnose and treat recurrent ACNES in a young and active patient. We describe a treatment-based diagnostic approach to confirm potential ACNES pain generators while recording pre- and post-treatment pain scores. After each maneuver, the patient was reassessed which allowed the working diagnosis to clinically evolve demonstrating the pathologic interrelationship between different skeletal structures and myofascial tissues contributing to irritation of the anterior cutaneous nerve. This treatment-based technique also made it possible to identify referred pain from a condition with overlapping symptoms originating from a different anatomic site. Treatment consisted of sequenced osteopathic manipulation techniques, active release techniques, instrument-assisted soft tissue mobilization, directional cupping, stretching, and strengthening exercises. The combination of sequenced treatments over the course of six physical therapy visits spanning 10 weeks resulted in 100% pain reduction and complete resolution of functional limitations. The patient was able to complete all work requirements and physical activity without pain. A sequenced treatment-based diagnostic approach to this case allowed us to more accurately identify all involved anatomic regions of pain and anatomic segments of pathology that were contributing to the abdominal pain or referring pain. No diagnostic imaging, invasive test, or injection was needed to properly diagnose and treat this case of ACNES. A proper understanding and application of osteopathic manipulation, active release techniques, instrument-assisted soft tissue mobilization, cupping, and exercises successfully resolved the contributing pain conditions and provided the patient important and useful tools and strategies to prevent recurrence.

## Introduction

Abdominal pain is a common condition frequently seen in the emergency department (ED) with a prevalence of 7-10% [1]. Given a broad differential diagnosis, it is difficult to accurately diagnose the cause of abdominal pain often requiring numerous, costly, potentially invasive, and ultimately unnecessary diagnostic imaging and tests [2]. Despite a lengthy work-up, some patients still leave the ED without a specific diagnosis or potentially a misdiagnosis. In one retrospective study, out of 3960 patients assessed for abdominal pain, 12.1% were misdiagnosed [3]. Chronic abdominal pain presenting to the ED is often incompletely worked up as approximately 52% of patients get discharged home without specific treatment; instead, they are given recommendations for referral to other medical services [1].

Exclusion, anterior cutaneous nerve entrapment syndrome (ACNES) is often underdiagnosed [4]. Approximately 2% of patients who present to the ED with abdominal pain have ACNES [5]. In ACNES, the peripheral nerve gets compressed as it traverses the posterior abdominal sheath resulting in focal abdominal pain when the abdominal wall contracts [6]. Pain then subsides when the patient lays supine and relaxes the abdominal muscles. Some symptoms of ACNES include hyperalgesia and pain to the skin in a specific area of the abdomen when touched or when pressure is applied digitally or with a pinch, and/or a positive Carnett’s sign. Carnett’s sign is positive when the index pain is reproduced when the patient contracts the abdominal muscles while the area of pain is digitally palpated [2,7].

The first-line treatment of ACNES is infiltration at the point of palpable pain with an anesthetic with or without a corticosteroid providing several weeks to months of relief [8,9]. Recalcitrant or chronic ACNES can be treated with repeated injections, pulsed radio-frequency ablation (PRFA), or surgical or chemical neurectomy involving removal or destruction of the painful nerve in the abdominal wall [6,10,11]. However, although these procedures often yield immediate results, a significant number of patients report temporary, partial, or no relief despite undergoing individual or a combination of the above invasive procedures [9,10]. While not uncommon, patients undergoing surgery can have surgical or postoperative complications and higher costs as compared to PRFA [10].

Partial or short-term response to invasive interventions may suggest that an undefined musculoskeletal impairment may be entrapping the nerve. The purpose of this case report is to describe a novel, conservative, non-invasive approach to evaluating and treating ACNES in a 21-year-old female. Second, we will highlight the interrelationship of different myofascial tissues directly resulting in irritation of the anterior cutaneous nerve and describe another condition whose pain referral pattern overlaps with symptoms of ACNES.

## Case presentation

A 21-year-old female presented with moderate to severe right lower quadrant abdominal pain of nine-month duration. She noted an insidious onset of abdominal pain four months following an uncomplicated vaginal delivery of her first child. She did endorse right-sided low back pain that started four months into her pregnancy but spontaneously resolved at eight months gestation. Her abdominal pain was rated as a constant 6/10 pain on the visual analog scale (VAS) but would increase to 8/10 with digital pressure to the right lower quadrant area, jogging, and lifting heavy objects. Lying supine also reproduced her pain to a level of 6/10. Over the nine-month period, her pain frequency and intensity gradually increased, despite being treated with anti-inflammatory medications and relative rest. Other pertinent past medical history included low back pain, right hip pain, headaches, and anemia. Labs including complete blood count and comprehensive metabolic panel were unremarkable. Abdominal computed tomography was normal. She had no known allergies and did not use tobacco products.

The patient was eventually referred to a pain management clinic by her primary care physician for a corticosteroid injection to treat presumptive ACNES. She was initially seen by the clinic’s physician assistant and was offered an injection; however, she elected to exhaust all conservative management options vice undergoing the injection. She was prescribed 200 mg celecoxib daily, instructed to avoid jogging, and referred to the clinic’s physical therapist.

Prior to the physical therapist’s exam, the patient completed the 36-Item Short Form Survey (SF-36) and the Defense and Veterans Pain Rating Scale (DVPRS) pain assessment tool. The SF-36 is a 36-item questionnaire that indicates the level of disability. Lower scores represent higher levels of disability along six sub-scales. The DVPRS assesses pain on a numeric scale from 0 to 10, and how the pain affects their daily activities, mood, sleep, and stress over the previous 24 hours. The SF-36 and DVPRS are both valid and reliable [12,13]. The patient’s scores for each visit are listed in Tables 1, 2.

**Table 1:** Standard Form-36 results reported during each visit

Visit (period since initial visit)	One	Two (1 week)	Three (2 weeks)	Four (3 weeks)	Five (7 weeks)	Six (9 weeks)
Physical functioning score	50%	50%	95%	90%	90%	100%
Role limitations due to physical health	75%	100%	100%	100%	100%	100%
Role limitations due to emotional problems	66.7%	100%	100%	100%	100%	100%
Energy/fatigue	75%	75%	85%	100%	90%	100%
Emotional well-being	92%	92%	88%	88%	84%	100%
Social functioning	25%	25%	100%	62.5%	50%	100%
Pain change	0%	35%	70%	45%	90%	100%
General Health	80%	100%	95%	90%	55%	100%
Health change	N/A	50%	100%	100%	100%	100%

**Table 2:** Defense and veterans pain rating scale results reported during each visit

Visit	One	Two (1 week)	Three (2 weeks)	Four (3 weeks)	Five (7 weeks)	Six (9 weeks)
Present pain level	6	1	1	0	0	0
In the past 24 hours with activity	5	1	1	1	2	0
In the past 24 hours affecting sleep	2	0	6	0	5	0
In the past 24 hours affecting mood	3	1	1	0	1	0
In the past 24 hours affecting stress	3	1	0	0	0	0

**Figure 1:**
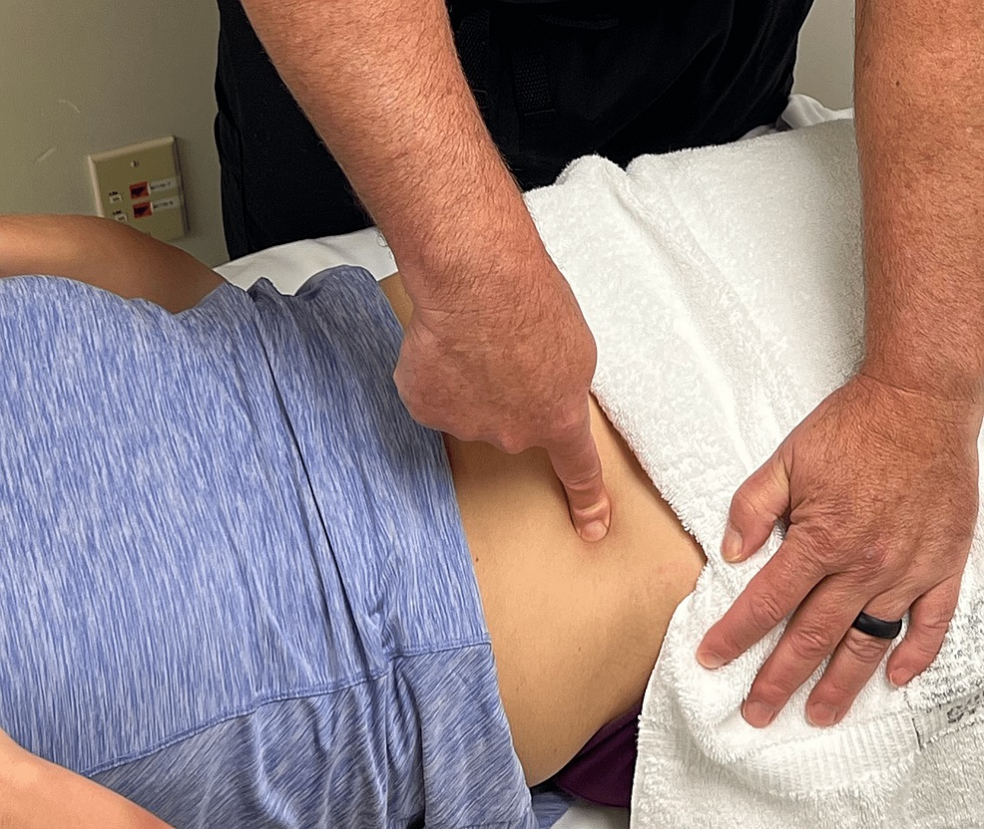
Carnette's Sign. Carnett’s sign is positive when the index pain is reproduced when the patient contracts the abdominal muscles while the area of pain is digitally palpated.

On physical examination, lumbar flexion to 50 degrees as measured with a bubble inclinometer placed on the L1 spinous process reproduced pain along the right posterior superior iliac spine (PSIS). No pain was reproduced with lumbar extension or side bending. Carnett’s sign was positive reproducing her index pain approximately two-inches medial and superior to the anterior superior iliac spine (ASIS) (Figure 1). While performing this test, the patient reported that her pain level was a 6/10. Digital palpation of the iliopsoas muscle just medial to the iliac crest also reproduced her abdominal pain. 

To confirm the potential pain generators, a sequenced treatment-based diagnostic approach was selected. Prior to treatment, testing for Carnett’s sign was performed, and the level of pain was recorded. Then a treatment maneuver was performed to address a specific biomechanical fault or tissue impairment. After each maneuver, Carnett’s sign was again assessed, and the pain response was recorded. The working diagnosis was ACNES, but the definitive diagnoses would evolve based on response to different interventions described chronologically over the 10-week treatment period. It was hypothesized that there was some interrelationship between her SIJ dysfunction and abdominal pain; therefore, both areas were treated concurrently.

Treatment

**Figure 2:**
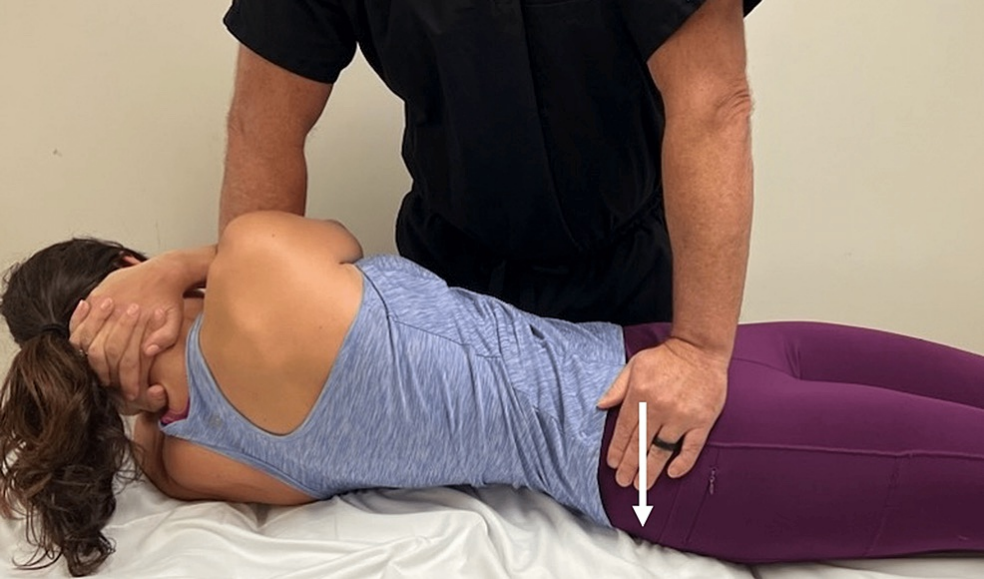
Sacroiliac joint osteopathic manipulation technique. The patient is placed in supine with hands clasped behind their neck. The examiner passively moves both legs and torso toward the right side. The torso is then rotated to the left fully, thereby locking the spine. The examiner’s caudal arm is placed through the patient’s arms to stabilize the torso while the other hand grips the right iliac bone. While the patient exhales, the examiner simultaneously rotates the torso farther while imparting a high-velocity, low-amplitude force through the ilium downward toward the table (white arrow).

The treatment plan consisted of a sequenced approach of osteopathic manipulation techniques (OMT), active release techniques (ART) to decrease muscle tension, instrument-assisted soft tissue mobilization (IASTM), directional cupping, stretching, and strengthening exercises. OMT techniques have been shown to be effective in combination with exercises for the treatment of SIJ dysfunction [14]. A posterior rotation force was applied to the right ilium with audible cavitation (Figure 2) [15]. 

**Figure 3:**
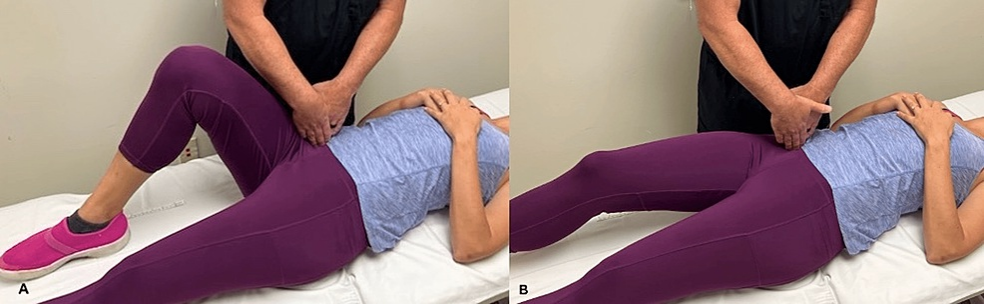
Iliopsoas active release technique. With the patient in supine, the provider places digital pressure just medial to the ASIS (A). While the pressure is maintained, the patient slowly straightens the leg while keeping the foot on the bed (B). Pressure is then released, while the patient brings the leg back to the starting position. This is repeated four times.

The patient was then able to fully flex forward without back pain, and while Carnett’s sign was positive, her abdominal pain level decreased to a 4/10. ART was applied to the right iliopsoas muscle just medial to the anterior portion of the iliac crest, and the patient actively straightened her leg (Figure 3). After four repetitions, the patient lay on her left side, and the right leg was held in a position to stretch the iliopsoas for 30 seconds. Repeat testing for Carnett’s sign was positive, but the pain level was rated as a 1/10. 

**Figure 4:**
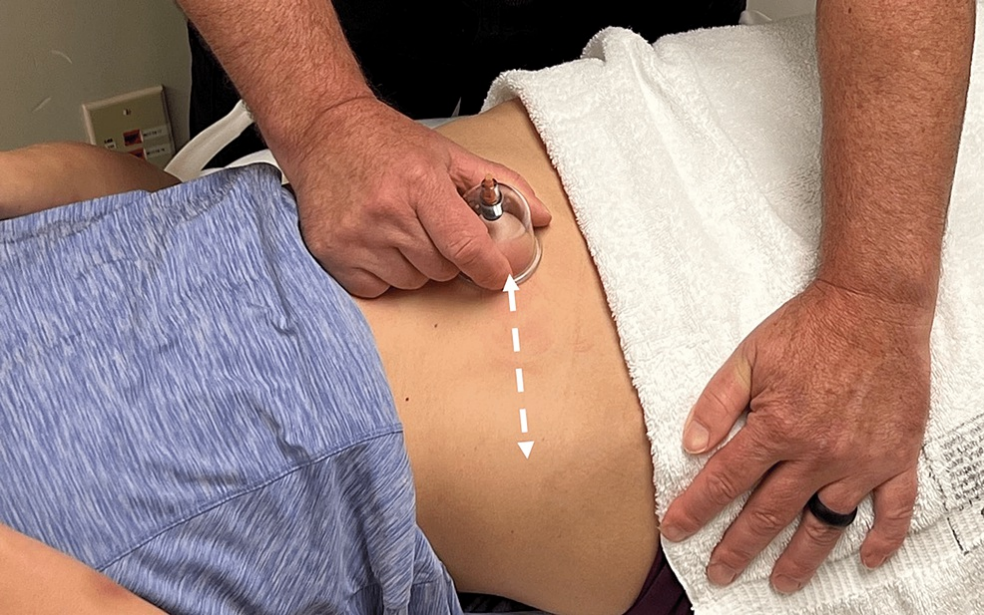
Directional cupping. After a lotion is applied to the skin, a 4 cm suction cup is applied lateral and inferior to the umbilicus at the point of index pain. Suction is applied and the cup is manually moved laterally along the course of the abdominal cutaneous nerve (white arrow). This is performed back and forth for 10 seconds and repeated four times after a period of rest.

Finally, directional cupping was performed. This technique involved lubricating the skin with lotion and placing a 4 cm vacuum suction cup over the area of pain. After suction is applied, the cup is manually moved laterally and then medially over the course of the anterior cutaneous nerve for three 10-second periods (Figure 4). After treatment, Carnett’s sign was negative.

The patient was provided instruction in daily stretching of the right iliopsoas and piriformis muscles and strengthening of the hamstrings, hip adductors, and abdominals using equipment at the gym every other day for one week. This treatment program was selected to treat the sacroiliac joint dysfunction [14]. By focusing on the SIJ joint, the authors hoped to better understand the potential interrelationship between SIJ dysfunction and abdominal pain or to determine if these were two separate conditions.

At the second clinic visit one week later, the patient reported that her pain level was a 1/10 (see Table 1). She had been performing her exercises consistently. To test her progress, she performed ballistic jumping exercises (AKA “Burpees”) the previous day which did not exacerbate her pain. She also reduced intake of celecoxib to 100 mg daily.

On exam, she demonstrated a full lumbar range of motion without back pain. Provocation testing of the SIJ was negative. With manual muscle testing of the iliopsoas, her abdominal pain was reproduced at a 4/10 level. Carnett’s sign was present at a level of 4/10. After ART to the iliopsoas, her abdominal pain decreased to a 1/10. No abdominal pain was reproduced following directional cupping. Immediately after treatment, she sprinted 20 yards without a return of her index back or abdominal pain. Prior to her follow-up one week later, she would jog one mile on a track.

 At the third clinic visit, the patient reported that she did jog one mile without prior stretching, and her abdominal pain level elevated to a 6/10. Her pain quickly resolved after stopping but did return to a 6/10 while she was lying in bed. The next day, her abdominal pain level was a 1/10. On exam, the resistance applied to the right iliopsoas elevated her pain level to a 6/10. Carnett’s sign was negative at the area of her initial abdominal pain but testing for Carnett’s sign was now positive one inch above and medial to the ASIS with a reported pain level of 9/10. Similarly, digital pressure to the iliopsoas muscle medial to the iliac crest reproduced her abdominal pain at a level of 9/10.

The sequence of treatment was changed to better understand which tissues were the primary pain generator. IASTM to the piriformis was performed for five minutes with a pneumatic vibration device (Vibracussor, Impacinc, Salem, Oregon) after which Carnett’s sign was negative. ART was applied to the right iliopsoas as the iliopsoas were still tight compared to the left side. Directional cupping over the course of the abdominal cutaneous nerve was continued to reduce the formation of additional adhesions. She was instructed to jog twice before her follow-up in one week, but she was to stretch the piriformis and iliopsoas prior to jogging. She had purchased a cupping set and performed the directional cupping treatment every two days at home.

At the fourth clinic visit, the patient reported only two episodes of abdominal pain lasting 10 seconds. She had been self-cupping every other day and noted that her pain only occurred on days in between cupping sessions. On exam, she denied any abdominal pain, but she noted posterior gluteal pain at a 2/10 level. Manual stretching of the piriformis reproduced her gluteal pain. Carnett’s sign was positive at a 1/10 level one-inch superior and medial to the ASIS, but not at the site of her index abdominal pain symptoms. IASTM to the piriformis resolved her gluteal pain, but Carnett’s sign was still present. With ART and manual stretching to the right iliopsoas, Carnett’s sign was negative. She was instructed to continue cupping every other day for one week and then discontinue that treatment. She would follow up in one month.

At the fifth clinic visit, the patient reported that she only experienced 2/10 level abdominal pain while jogging and 5/10 while lying down. She noted that while jogging, both her abdominal and gluteal pain were present. Carnett’s sign was present at a 2/10 pain level. After ART to the iliopsoas, Carnett’s sign was still positive, but after IASTM to the piriformis, it was negative. She was instructed to focus on piriformis stretching prior to jogging. As her index nerve entrapment symptoms were no longer present, cupping was not performed. It was hypothesized that the piriformis tightness was referring to pain in the lower quadrant or causing some biomechanical fault resulting in a pathologic load upon the anterior abdominal cutaneous nerve somewhere along the nerve path. She would follow-up in two weeks.

At the sixth visit, the patient reported no abdominal pain or gluteal pain. Her rated pain was 0/10 and there was no disability along the SF-36’s six subsets (Tables 1, 2). She was able to complete all the physical requirements for her job. She was discharged from the pain management clinic. At one year post-treatment, she was in the third trimester of her second pregnancy and reported no recurrence in her index abdominal and low back pain.

## Discussion

In patients presenting with chronic abdominal pain, it can be difficult to develop a specific diagnosis given a broad range of differential diagnoses requiring a lengthy and costly work-up. Often overlooked initially, ACNES can mimic functional and structural gastrointestinal disorders. Neuromusculoskeletal causes of abdominal pain are not considered until functional gastroenterologic disorders are ruled out [2,16]. Musculoskeletal disorders that have symptoms that overlap with ACNES include thoracic disc herniation and rectus abdominus strain or tear while neuroanatomic conditions that refer pain to the lower quadrant include thoracic radiculopathy and iliohypogastric or intercostal nerve entrapment. In 2% of patients diagnosed with sacroiliac joint pain verified with a diagnostic injection, their abdominal pain subsided indicating that the sacroiliac joint pathology can refer pain to the abdomen [17].

There are several possible etiologies and shared characteristics of ACNES. In a retrospective cohort study of 139 patients diagnosed with ACNES via an anesthetic block, 54% of the patient’s symptoms were spontaneous while 20% were believed to be related to previous surgery and 9% from pregnancy [8]. However, in most cases of idiopathic ACNES, there is no understood cause [11}. Mol et al. (2021) noted that most of the patients in their cohort of 1116 consecutive patients were female, either young or middle-aged, and with a normal BMI [7]. The onset of symptoms was spontaneous and progressed in severity and chronicity after delayed definitive care [7]. In our case, our patient shared many of these same characteristics.

Once ACNES is confirmed, treatment options include repeat injections with corticosteroids, PRFA, and surgical or chemical neurectomy [8,10,11]. Comparative studies of documented modalities suggest some benefit with each; however, the technique for injections (ultrasound-guided vs. anatomical landmark-guided) and patient response to surgical factors (surgical site pain and risks associated with surgery including bleeding and infection) make equivalent comparisons challenging. Determining a clinically significant percentage reduction in pain depends on numerous variables, such as starting pain intensity, the extent of prior surgery, attributes of the pain, patient-reported outcomes, etc., and as such, there is no universally accepted standard for percentage pain reduction defining a “positive” outcome following a procedure. In the cited studies, as well as in our clinical practice, positive outcomes are defined as 50% reduction in pain score post-procedure. While a VAS pain score may not meet the 50% improvement threshold, satisfactory patient-reported functional improvement with certain aggravating activities may also be considered treatment success. Although both injection and neurectomy groups experienced measurable pain relief, the duration of this benefit varied across the literature based on the modality used with local anesthetic and steroid injection demonstrating a shorter duration of pain relief whereas surgical/chemical neurectomy demonstrated a longer duration of pain relief [8]. While some patients may inexplicably report permanent 100% pain relief after a single diagnostic injection with a short-acting local anesthetic [8], rarely is any single modality the definitive solution. 

After a review of the literature, there is only one case study describing a non-operative management program in a patient with persistent ACNES two years following abdominal surgery [18]. To our knowledge, there are no documented studies that outline a non-invasive treatment approach in patients with idiopathic ACNES who did not undergo surgery. This case report emphasizes the high-yield physical exam findings and proposes a viable non-invasive (injection or surgery) option to link possibly interrelated myofascial tissues and biomechanical faults and offers a novel treatment for ACNES with or without the presence of sacroiliac joint dysfunction. If two percent of patients with SIJ dysfunction have referred pain to the abdomen combined with the fact that the literature supports ACNES being significantly underdiagnosed, there exists a potentially large percentage of patients (both adults and children) who may be undergoing unnecessary diagnostic exams and invasive procedures. 

At presentation to the pain management clinic, the patient had previously been diagnosed with presumptive ACNES. Anatomically, the anterior cutaneous nerve is a branch of the anterior rami of the 7th-12th intercostal nerves which are sensory nerves that travel between the transversus abdominus and internal oblique muscles to the rectus sheath. At the posterior rectus sheath, the nerve turns 90 degrees and enters a neurovascular bundle surrounded by a fibrous ring or freely between planes of the posterior rectus sheath and eventually reaches the skin [7,10]. It is postulated that the entrapment occurs within this fibrous ring resulting in neuroischemic pain [10]; however, there may be other pathophysiological mechanisms as 60% of ACNES is idiopathic [8].

In our case, because the patient had a history of and presented with low back pain in addition to her right lower quadrant pain, it was hypothesized that the nerve entrapment may have been exacerbated by the SIJ dysfunction and its associated impairment pattern rather than as an isolated condition. Typically, pain should improve when the patient lies down and relaxes the abdominal muscles. In this case, one of the aggravating factors was an exacerbation of pain when she was lying supine. Such a symptom could exclude a diagnosis of ACNES.

Given the overlapping back and abdominal symptoms, a treatment-based diagnostic approach was applied to both areas sequentially. The sacroiliac joint was first manipulated resulting in the resolution of her back pain and improvement of her tolerance to digital pressure applied to the area of her index abdominal pain. This immediate reduction in both back and abdominal pain corresponded to SIJ pain referral patterns described by Slipman (2000) but did not completely rule out nerve entrapment [17]. Similarly, addressing muscular tightness (i.e., piriformis and iliopsoas) associated with SIJ dysfunction on the first and second visits [15], also reduced her abdominal pain with repeated testing for Carnett’s sign. 

The last modality applied was directional cupping. This technique resulted in complete but temporary resolution of her abdominal pain on the first visit. While the exact mechanism of action has not been identified [19], we believe that cupping disrupts suspected adhesions between the surface of the skin and the anterior abdominal sheath. We would also postulate that the suction may be mobilizing the nerve through the ring by pulling the distal portion of the nerve as the cup is moved over the skin. 

By the third and fourth visits, the area of pain had lateralized by one inch laterally and inferiorly from the area of her index pain. Lateralization of pain has been demonstrated in 9% of patients following neurectomy and suggests that the nerve entrapment may have occurred at the level of the lateral margin of the posterior rectus sheath [8]. In our case, we postulated that the digital pressure could be loading the iliopsoas muscle as it passes under the new area of pain potentially making the iliopsoas an interrelated pain generator. By applying resistance as the patient contracted the iliopsoas, her pain level rose to a 6/10. To reduce muscle tone, an active release technique was utilized (Figure 3) followed by manual stretching of the muscle. After the technique, Carnett’s sign was negative.

Over the next 30-day period, the patient experienced only two brief episodes of pain spontaneously. As we believed the entrapment could not have resolved after only two sessions of tissue mobilization along the course of the nerve, she continued the cupping regimen every other day for one week. Her abdominal pain was only experienced with jogging and most interestingly when she was lying in bed. We suspect that these findings could be explained by continued tightness of the iliopsoas, but during treatment, there was no change in her pain level with testing for Carnett’s sign following active release and stretching of the iliopsoas. Instead, we performed instrument assisted deep tissue mobilization to the piriformis which resulted in a negative Carnett’s sign. This result contributes to our belief that ACNES and tissue impairments commonly associated with SIJ dysfunction may be inter-related and should be assessed concurrently.

Given the delay in definitive care and high costs associated with potentially inconclusive diagnostic testing for non-specific abdominal pain [12], we suggest that the diagnostic work-up and referral to a provider that can perform osteopathic manipulation to the SIJ and soft tissue mobilization should be done in parallel. If the treating provider can reproduce and subsequently decrease the index abdominal pain with the approach as described above, diagnostic testing (i.e., CT, ultrasound) can be curtailed. Similarly, this approach may be an appropriate alternative for patients not wishing to undergo injections or neurectomy. For those who elect to undergo invasive treatment but do not receive adequate relief, sacroiliac joint dysfunction and myofascial tightness should be considered as potential pain generators.

The current case cannot be generalized to all cases; however, given the patient’s remarkable improvement, future research to identify the incidence of comorbid sacroiliac joint dysfunction and ACNES would be helpful. A future randomized controlled study could compare isolated nerve block versus osteopathic manipulation and soft tissue mobilization versus both combined. Additional research could also compare physical therapy performed in a sequential treatment-response-based algorithm, as demonstrated in this case, to a focal anatomic approach confined to a single suspected diagnosis. We postulate that a sequential treatment-response-based algorithm is more effective and further research may direct a paradigm shift to broaden physical therapy assessment and adjust treatment protocols.

## Conclusions

Abdominal pain is a common problem for people of all ages. Due to the wide range of differential diagnoses, it is difficult to determine the exact etiology. ACNES is one of the differential diagnoses that causes chronic abdominal pain and often goes overlooked in the ED setting. This case report suggests that osteopathic manipulation and soft tissue mobilization can be a safe and effective treatment for ACNES. After functional gastroenterological disorders have been ruled out, a referral for this therapy should be considered to confirm the diagnosis and minimize a more lengthy and costly evaluation.
